# Evidence for a visual bias when recalling complex narratives

**DOI:** 10.1371/journal.pone.0249950

**Published:** 2021-04-14

**Authors:** Rebecca Scheurich, Caroline Palmer, Batu Kaya, Caterina Agostino, Signy Sheldon

**Affiliations:** Department of Psychology, McGill University, Montreal, QC, Canada; University of St Andrews, UNITED KINGDOM

## Abstract

Although it is understood that episodic memories of everyday events involve encoding a wide array of perceptual and non-perceptual information, it is unclear how these distinct types of information are recalled. To address this knowledge gap, we examine how perceptual (visual versus auditory) and non-perceptual details described within a narrative, a proxy for everyday event memories, were retrieved. Based on previous work indicating a bias for visual content, we hypothesized that participants would be most accurate at recalling visually described details and would tend to falsely recall non-visual details with visual descriptors. In Study 1, participants watched videos of a protagonist telling narratives of everyday events under three conditions: with visual, auditory, or audiovisual details. All narratives contained the same non-perceptual content. Participants’ free recall of these narratives under each condition were scored for the type of details recalled (perceptual, non-perceptual) and whether the detail was recalled with gist or verbatim memory. We found that participants were more accurate at gist and verbatim recall for visual perceptual details. This visual bias was also evident when we examined the errors made during recall such that participants tended to incorrectly recall details with visual information, but not with auditory information. Study 2 tested for this pattern of results when the narratives were presented in auditory only format. Results conceptually replicated Study 1 in that there was still a persistent visual bias in what was recollected from the complex narratives. Together, these findings indicate a bias for recruiting visualizable content to construct complex multi-detail memories.

## Introduction

Recalling complex events, including narratives or personal experiences, requires constructing a detailed mental representation by binding together the associated details of those events [1–3]. That these representations are constructed suggests that not all details associated with a memory are retrieved the same way. While some details will be remembered as they occurred, others will be forgotten and left out of that representation or recalled in a distorted manner. It is not yet clear if different types of details contained in complex events, whether auditory, visual, or non-perceptual, are subject to different fates during recall. That is, does the nature of an event detail within a complex memory determine if it is to be remembered, forgotten, or distorted? Based on prior work indicating that visual details are preferentially recruited to form complex memory representations, perhaps due to easy encoding and reactivation of visual information [4], we hypothesized that complex narratives presented with visualizable details will be recalled more accurately than the same complex narrative presented with non-visual perceptual content. We hope to provide insight into the biases that exist in encoding and retrieving complex memories.

If the type of perceptual content determines the fate of recall, it could be that different forms of memory are responsible for encoding and retrieving different types of content. Research has proposed that information from complex events can be represented as gist or as verbatim memory. Gist memory is when the underlying meaning or content of presented information is used to represent that information within a memory. Verbatim or detailed memory is when the underlying meaning as well as the specific superficial qualities that were present at encoding are used to represent that information within a memory [5]. According to Fuzzy Trace Theory [6], using gist processing when encoding information leads to that information being represented more abstractly when later constructing a memory representation. This makes gist-based information more susceptible to forgetting and memory distortions. In contrast, engaging in verbatim memory involves storing and recalling the precise sensory details of that retrieved information, therefore leading to more accurate memory recall. In other words, a complex memory formed without specific perceptual inputs presented during encoding is more likely to be subject to distortions at retrieval.

These forms of memory can be dynamically engaged, at least in younger adult populations, which suggests that there are certain types of memories that preferentially engage a gist versus verbatim form of memory [7]. One factor that makes engaging in verbatim memory more likely is the presence of imageable details. That is, the likelihood of using verbatim memory for encoding, storing, and retrieving complex memories is a function of the ability or ease of constructing the memory representation. Prior work has suggested that this ability depends on how strongly imagery processes are activated when processing information, with most of this work done exclusively in the visual domain. Research has shown that the visual imageability of a word, or the extent to which it evokes a mental image, determines how well that word is recalled [8]. Furthermore, several tests have shown that visual imagery can predict recall accuracy for standard stimuli like word lists [9]. However, whether the visual imageability of more complex and naturalistic information engages verbatim memory in the same way is an open question.

Autobiographical memory research, which can be considered a natural and complex type of episodic memory, suggests that visual imagery is used to retrieve sensory-perceptual details of a memory, which underscore verbatim and accurate recall [10, 11]. Mental representations of complex autobiographical events, which engage vivid visual perceptual processes, are recalled more richly [1, 4, 12], raising the question of whether the benefit of recalling a visually rich complex event extends beyond the visual elements of that event. This question relates to the hypothesis that visual information can act as a mental image scaffold for retrieving other components of a constructed event representation [13]. In accordance with this hypothesis, previous findings have shown that visual imagery processes, when engaged while constructing mental representations of past episodes, are activated to help remember other non-visual event content [14–16]. Thus, it is plausible that the ability to accurately recall visual information within a complex event, through verbatim memory, will extend to other details within a complex memory.

If the impact of visual information on verbatim recall of complex episodic events is through imagery, it is important to consider if other perceptual details that could activate imagery processes also evoke verbatim memory. Another form of perceptual information within complex memory known to stimulate imagery processing is auditory information. There is a limited amount of research focusing on how auditory details are represented in episodic memories, particularly those that represent real-world scenarios [e.g., 17–19]. This research suggests that auditory dimensions are simulated during retrieval of auditory memories [20]. Furthermore, retrieval of auditory memories involves reactivation of brain regions specific to the stimulus being recalled, such as those involved in speech processing when recalling words [21]. While this work might suggest that auditory information should also evoke verbatim memory processing, whether this information can do so as well as visual information is not known. We examine this question in the current studies.

### Current research

There are open questions about how perceptual details are recalled from complex memories of events that are experienced in everyday life. One primary question is whether visual and auditory details from the same episodic memory are recalled differently. Although visual and auditory information are processed differently [22, 23], it is not clear how these forms of information are recollected when they interact in more complex and naturalistic scenarios. To study this question, we used an experimental paradigm in which participants recollected stimuli that reflected the complexity of naturalistic and autobiographical memories–everyday narratives—to determine the accuracy with which different types of information in these narratives were recalled. Using these naturalistic-like stimuli in which it was known what information was being encoded further allowed us to examine how that information was recalled, with gist or verbatim memory. Our primary hypothesis was that information represented with visual details would be more likely to be encoded and recalled with verbatim memory by virtue of the ability to imagine these details within the event’s memory representation. We also hypothesized that information represented with auditory details would be more likely to be encoded with gist memory, leading to less accurate recall, because these details are less imageable. Following the principles of Fuzzy Trace Theory [6], this reduction in accuracy would present as both auditory details being forgotten at a greater rate than visual details and these details being distorted during recall.

To test these hypotheses, we conducted two studies. In Study 1, we presented videos to participants in which an actress described personal events through visual, auditory, and non-perceptual information. Multiple versions of these event narratives were created where the general content of the details was held constant while the perceptual content (i.e., visual and auditory) was manipulated. This allowed us to directly compare how the perceptual description of the same information affected memory when participants later recalled these narratives. To this end, we examined whether the perceptual (visual and auditory) and non-perceptual details were retrieved in a gist-based manner or with specific story content, representing verbatim memory. Based on previous research suggesting that encoding and retrieving visual information from episodic memory involves verbatim memory, we predicted that memory would be most accurate for the narratives described using primarily visual information. We further predicted more memory distortions, indicative of reduced verbatim memory, for the narratives described using primarily auditory information. We aimed to conceptually replicate this finding in Study 2, in which participants studied the same narratives in audio only format and with the narratives segmented into chapters to reduce task difficulty.

## Study 1

### Methods

#### Participants

Forty-one participants were recruited, however one was excluded due to insufficient data from the free recall phase of the study. Thus, the analyzed sample included 40 English speakers (mean age = 21 years old, SD = 2.06 years; 29 female; one additional participant excluded from demographics due to missing data) recruited through McGill University’s participant pool and the Montreal community to participate in this study. All participants reported normal hearing. Those recruited through the Montreal community were given a small compensation for their participation, and those recruited through the participant pool were given course credit.

#### Stimuli and experiment materials

Six narratives were selected from stimuli used by [24]. These narratives were adapted to create a visual, an auditory, and a multimodal version of each one, resulting in 18 total narratives (6 in each Visual, Auditory, and Multimodal Narrative Condition). All narratives included the same number of details (56) and the same number of sentences across different versions of each narrative (Table 1). Twelve of these details were described with non-perceptual content (hereafter referred to as neutral details) that were presented identically across versions. The remaining 44 details were described with different perceptual content across versions. For the visual narratives, all 44 details were described with visual information. For the auditory narratives, all 44 details were described with auditory information. For the multimodal narratives, 22 details were described with visual information and 22 details were described with auditory information. In other words, the perceptual content present in each narrative changed while the non-perceptual content forming the overarching storyline of the narrative remained constant. An additional multimodal narrative that was of similar length was created to be used as a practice stimulus in the experimental task.

**Table 1:** Word and sentence counts per story and condition.

*Story Title*	*Condition*	*Word Count*	*Sentence Count*
	Visual	470	39
*Trip to the Beach*	Auditory	512	39
	Multimodal	489	39
	Visual	540	44
*Flight to London*	Auditory	566	44
	Multimodal	531	44
	Visual	567	44
*Engagement Party*	Auditory	553	44
	Multimodal	536	44
	Visual	537	38
*Camping*	Auditory	541	38
	Multimodal	524	38
	Visual	567	38
*The Garden*	Auditory	674	38
	Multimodal	582	38
	Visual	640	41
*San Francisco*	Auditory	734	41
	Multimodal	657	41

A female actress (20 years old, native English speaker) was recruited to perform each narrative, which was video recorded. The actress was seated in front of a green screen for each video recording and was instructed to perform the narratives as if she were describing her own past personal events (i.e., conversationally). Written informed consent was obtained from the actress to present the videos recorded from this session (3–5 minutes each) to participants. None of the participants in this study reported knowing the actress.

The videos were presented individually to participants audiovisually via a Dell computer running Windows 7. Participants’ free recall of the videos was recorded using Olympus WS-852 and Sony ICD-PX333 voice recorders. Participants’ subjective ratings of videos and responses to questionnaires during the delay phase were collected using Eprime 2.0.

#### Design

The study used a within-subjects design with the independent variables of Narrative Condition (Visual, Auditory, and Multimodal) and Detail Type (Visual, Auditory, and Neutral). The dependent variables were the proportion of details recalled with the correct gist (gist-based memory), the proportion of details recalled with the correct perceptual content (verbatim memory), and the proportion of details recalled with the incorrect perceptual content (false recollections; see **Data Analysis**). Each participant was presented with all 6 narratives (2 from each Narrative Condition). The Narrative Condition assigned to each narrative as well as the narrative presentation order was randomized for each participant.

#### Procedure

All procedures were approved by the McGill University Research Ethics Board and written informed consent was obtained from all participants upon arrival to the lab. Participants were instructed that they would be viewing several videos of someone describing her past personal events, and that they would be asked to recall as much as possible from these events later in the study. They were then presented with a practice phase of the study to become familiar with this task. Participants were given an audiovisual presentation of the practice video title (i.e., “Barb works at a store”) via headphones and a computer screen before and after viewing the corresponding video. After the final presentation of the audiovisual title, participants were then asked to verbally recall as much as possible from the video. Participants then moved on to the study phase in which they viewed all six experimental videos. As in the practice phase, participants were given an audiovisual presentation of each experimental video title (e.g., “Barb waits for a flight”) before and after viewing the corresponding video.

After viewing all experimental videos, participants had a delay period (approximately 20 minutes) prior to the test phase in which they completed a series of questionnaires that were unrelated to the narrative content and not examined in this study.

**Fig 1:**
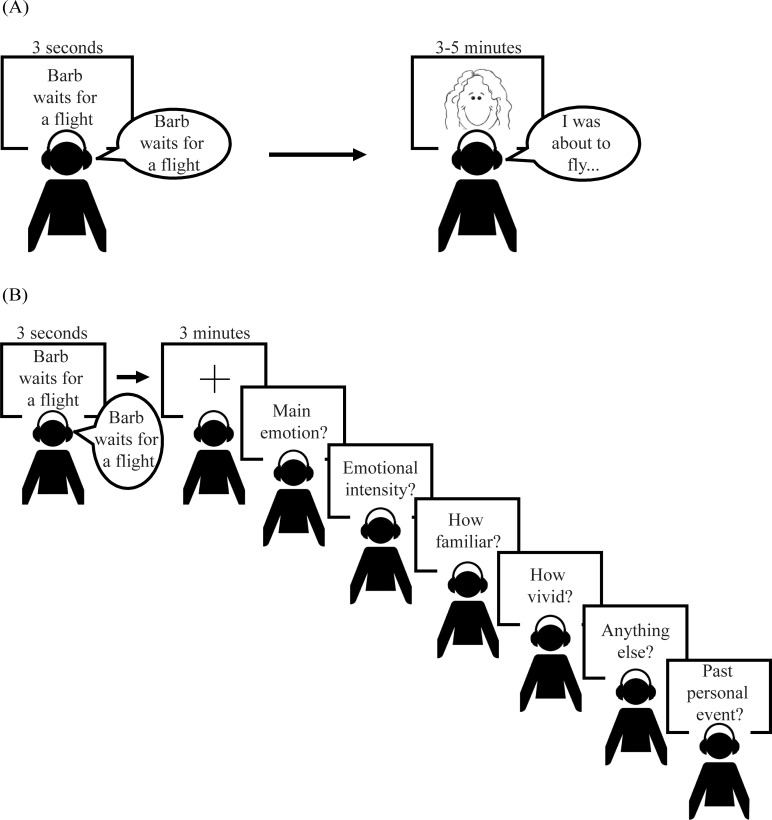
Visual depiction of procedure in Study 1. (A) Study phase procedure: Participants were presented with the narrative title visually for 3 seconds while also hearing the narrative title spoken by the actress. Participants then watched and listened to the corresponding video which lasted between 3–5 minutes. (B) Test phase procedure: Participants were presented with the same audiovisual title as in the study phase and were then given 3 minutes to verbally recall as much as possible from the corresponding narrative. Follow free recall, participants were asked to indicate the narrative’s main emotion, emotional intensity, familiarity, vividness, if there was anything else they could recall, and whether the narrative reminded them of a past personal event.

During the test phase, participants were presented with the audiovisual title of each narrative and then were given up to three minutes to freely recall as much as possible from the corresponding narrative. At the end of three minutes, participants rated each narrative for its emotional valence (1-negative, 2-neutral, or 3-positive), emotional intensity (1-not at all to 6-very intense), how vividly they could imagine that narrative (1-not vivid at all to 6-very vivid), and familiarity (1-not familiar at all to 6-very familiar). They were then given a general probe asking if there was anything more they could recall from each narrative, and if it reminded them of a past personal event. Fig 1 summarizes the procedure for the study and test phases.

#### Scoring

Participants’ recall descriptions were first transcribed (blind to condition) and subsequently scored for the presence or absence of a narrative detail, first at the gist level (presence of the general content) and then at the verbatim level (presence of the same perceptual content–visual, auditory, or neutral–as the narrative). Notably, a correct score for gist and verbatim memory indicated a detail recalled as it was presented. However, a correct score for gist and an incorrect score for verbatim memory could indicate that the person forgot the perceptual content or distorted the perceptual content during recall. Thus, we further coded verbatim memory in terms of whether perceptual content was recalled, but in a mismatched state. For example, the detail “I heard him turn on his lawn mower to mow the grass,” could be recalled matched in perceptual content, “She *heard* her neighbour mowing his lawn,” indicating correct gist and verbatim memory. It could be recalled without perceptual content (i.e., only a gist memory), “Somebody was working on their lawn.” Or it could be recalled mismatched in perceptual content, “She *saw* her neighbour mowing his lawn,” indicating a memory distortion. In the last case, the narrative detail was presented with auditory information but recalled with visual information. Importantly, to avoid subjective judgments by the part of the rater in scoring each detail, details were only given a perceptual (i.e., non-neutral) assignment by raters if visual or auditory descriptors were explicitly said by participants. This could take the form of the examples above (e.g., “She *heard* her neighbour mowing his lawn”), or by using the exact visual or auditory descriptors from the original narratives (e.g., “The whole city became *quiet*”).

In the scoring of transcriptions, a few additional steps were taken. If participants recalled details that were not present in the story, these additional details were not scored. If participants gave commentary on the story during their free recall, this was also not scored. Importantly, free recall scoring was adapted to the speaking style of each participant. This ensured that participants who frequently used perceptual descriptors (e.g., “see”) in their speaking, reflecting their manner of speech rather than a true assignment of perceptual content, were not artificially assigned the corresponding perceptual content for their free recall. For example, perceptual descriptors used as filler words or in expressions (e.g., “You see, Barb thought she heard the neighbour’s lawn mower.”) were not scored.

Participants’ free recalls were scored by a single rater. The rater was aware of the condition of each free recall as matching descriptions and perceptual content to that in the original narrative required knowledge of the narrative condition.

#### Data analysis

From the free recall scoring, we calculated three proportions to address our predictions. First, we calculated a gist memory score, which was the number of details recalled from the narrative, both neutral and perceptual, over the total details within the narrative. A detail was considered as recalled with gist memory if the overall concept or idea of the detail was recalled correctly (e.g., the detail “I was eating a croissant for lunch” was recalled as “She ate lunch”). We then calculated verbatim memory scores, separately for neutral and perceptual details, as the number of these details that were recalled with the same specific content as the narrative (e.g., the detail “I heard my neighbour turn on his lawn mower” was recalled as “She heard a lawn mower”) over the total number of those types of details within the narrative. Of note, a perceptual detail was only scored as recalled with verbatim memory if it included the correct percept (visual or auditory). Finally, we calculated false recollections scores which focused on details recalled with gist but not verbatim memory to examine how the original perceptual content of the detail was falsely recalled in these cases. We first calculated the number of details falsely recalled with visual information (e.g., a detail presented with auditory or neutral information that was recalled with visual details absent at encoding, such as the detail “I heard a dog bark” recalled as “She saw a dog”). We next calculated the number of details falsely recalled with auditory information. We then calculated the proportion of falsely recalled details with visual or auditory information over the total number of details that could be falsely recalled within the narrative (e.g., number of auditory and neutral details for visual false recollections). One detail in a multimodal narrative was miscoded for perceptual content and was therefore excluded from scoring and analysis.

#### Data analysis

Repeated measures Analyses of Variance (ANOVAs) were conducted on the memory scores, with Narrative Condition as the repeated factor and Detail Type (verbatim memory) or Misremembered Detail Type (false recollections) as an additional factor when necessary. ANOVAs were also run on participants’ subjective ratings of emotional valence, emotional intensity, vividness, and familiarity, with Narrative Condition as factor, to ensure that any observed effects were not due to differences in the way the narratives were experienced at recall. To follow up significant main effects and interactions, post-hoc *t*-tests were conducted using the Holm correction method [25].

### Results

#### Ratings

The one-way repeated measures ANOVAs on each subjective rating showed no significant main effect of Narrative Condition for any of the subjective ratings (emotional valence: *F*(2, 76) = 0.49, *p* = 0.61; emotional intensity: *F*(2, 76) = 0.11, *p* = 0.89; vividness: *F*(2, 76) = 0.05, *p* = 0.96; familiarity: *F*(2, 76) = 2.00, *p* = 0.14; one participant was excluded from this analysis due to missing data). This confirmed that observed effects were not due to differences in participants’ subjective ratings across conditions.

#### Gist memory

**Fig 2:**
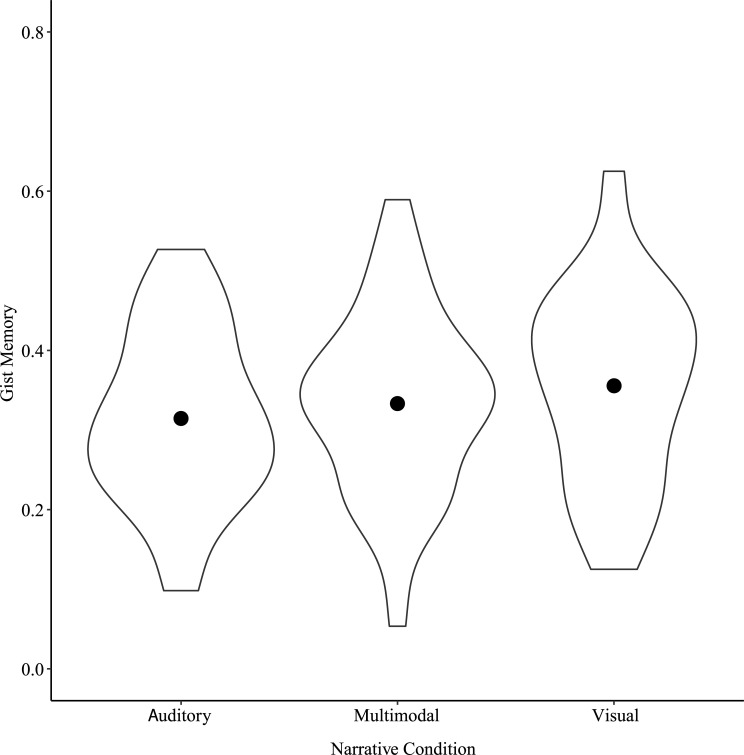
Gist memory by Narrative Condition for Study 1. Points show means for each Narrative Condition.

A one-way repeated measures ANOVA on gist memory scores with Narrative Condition as factor showed a significant main effect of Narrative Condition, *F*(2, 78) = 5.95, *p* < 0.01, η^2^_p_ = 0.13 (Fig 2). Post-hoc tests showed a significant difference between Auditory and Visual narratives, *t*(78) = -3.45, *p* < 0.01. The gist memory score was greater for Visual than for Auditory narratives.

#### Verbatim memory

**Fig 3:**
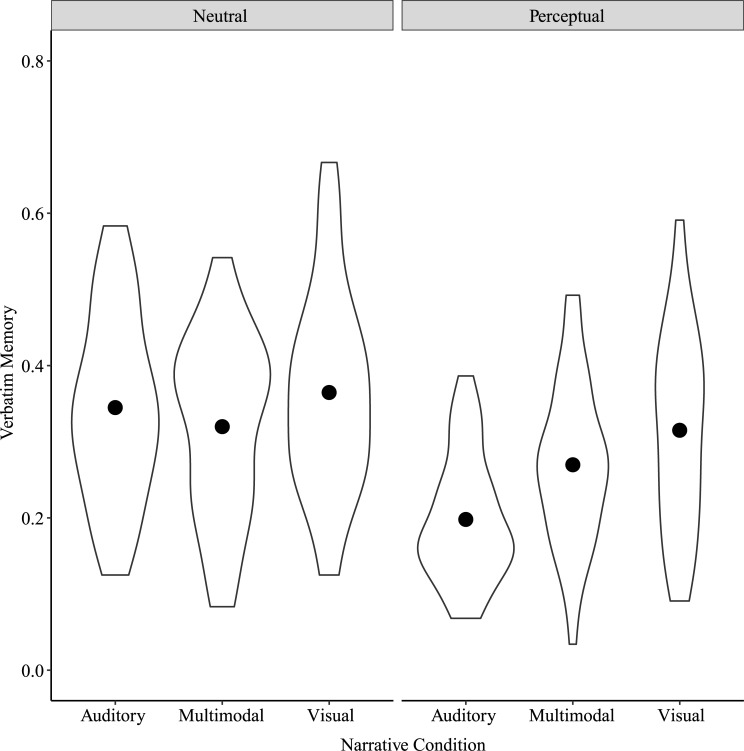
Verbatim memory by Narrative Condition and Detail Type for Study 1. Points show means for each Narrative Condition by Detail Type.

Because we were interested in whether verbatim memory was engaged in different ways depending on the specific perceptual content of the details, we ran a two-way repeated measures ANOVA on verbatim memory scores with Narrative Condition and Detail Type (Perceptual or Neutral) as factors. Results showed significant main effects of Narrative Condition, *F*(2, 78) = 17.10, *p* < 0.001, η^2^_p_ = 0.31, Detail Type, *F*(1, 39) = 43.70, *p* < 0.001, η^2^_p_ = 0.53, and a significant interaction between Narrative Condition and Detail Type, *F*(2, 78) = 14.20, *p* < 0.001, η^2^_p_ = 0.27 (Fig 3). Post-hoc tests showed significant differences in verbatim memory for Perceptual details between Auditory and Multimodal narratives, *t*(154) = -4.52, *p* < 0.001, between Auditory and Visual narratives, *t*(154) = -7.37, *p* < 0.001, and between Multimodal and Visual narratives, *t*(154) = -2.85, *p* = 0.04. For Perceptual details, these scores were greatest for Visual narratives, followed by Multimodal and then by Auditory narratives. For Neutral details, these scores differed only between Multimodal and Visual narratives, *t*(154) = -2.82, *p* = 0.04.

#### False recollections

**Fig 4:**
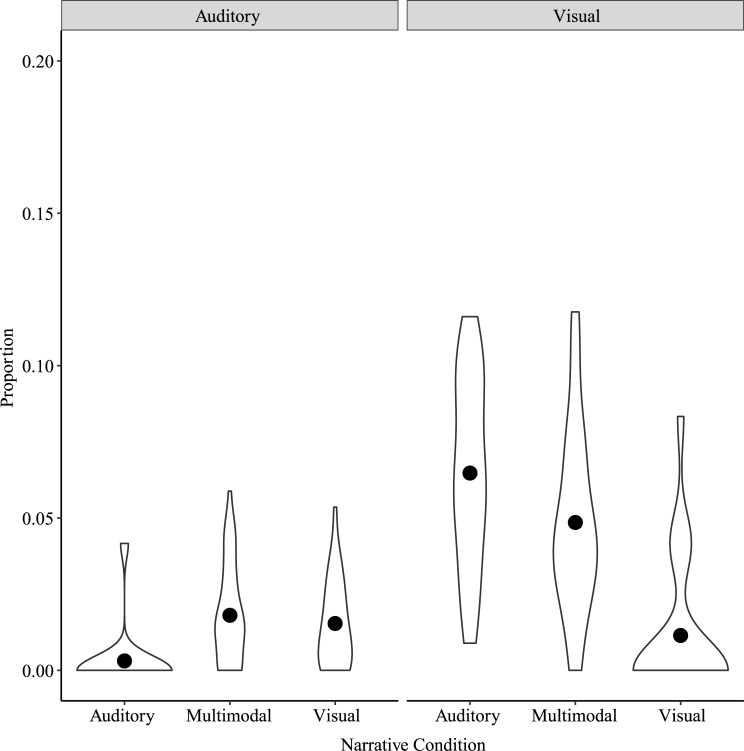
False recollections by Narrative Condition and Misremembered Detail Type for Study 1. Points show means for each Narrative Condition by Misremembered Detail Type. Note the y-axis scale, indicating very few false recollections.

In examining the false recollection score, we focused on Visual and Auditory (i.e., Perceptual) false recollections to see how the specific perceptual content influenced memory distortions. A two-way repeated measures ANOVA on false recollections with Narrative Condition and Misremembered Detail Type as factors showed significant main effects of Narrative Condition, *F*(2, 78) = 25.20, *p* < 0.001, η^2^_p_ = 0.39, Misremembered Detail Type, *F*(1, 39) = 123.00, *p* < 0.001, η^2^_p_ = 0.76, and a significant interaction between Narrative Condition and Misremembered Detail Type, *F*(2, 78) = 44.10, *p* < 0.001, η^2^_p_ = 0.53 (Fig 4). Post-hoc tests showed significant differences for Visual Misremembered Detail Type between Auditory and Visual narratives, *t*(155) = 11.11, *p* < 0.001, Auditory and Multimodal narratives, *t*(155) = 3.38, *p* = 0.01, and Multimodal and Visual narratives, *t*(155) = 7.73, *p* < 0.001. These Visual Misremembered Details were greatest for Auditory narratives, followed by Multimodal narratives and then by Visual narratives. For Auditory Misremembered Detail Type, post-hoc tests only showed a significant difference between Auditory and Multimodal narratives, *t*(155) = -3.12, *p* = 0.01. Auditory Misremembered Details were greater for Multimodal narratives than for Auditory narratives. There was no significant difference between Visual and Multimodal narratives or between Visual and Auditory narratives.

**Fig 5:**
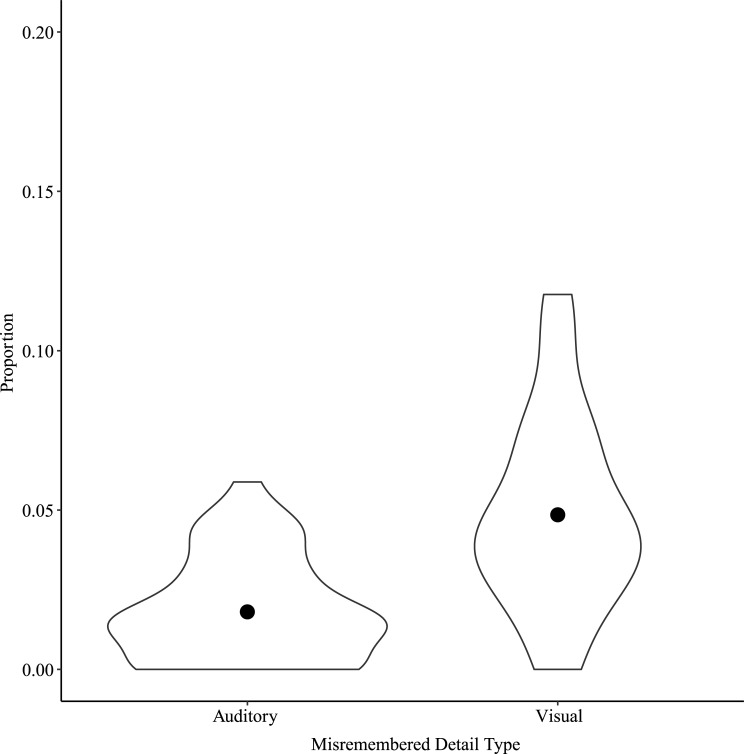
False recollections by Misremembered Detail Type for the Multimodal Narrative Condition in Study 1. Points show means for each Misremembered Detail Type in the Multimodal Narrative Condition. Note the y-axis scale, indicating very few false recollections.

To address the possibility that the observed pattern of results was due to a different number of opportunities for Visual and Auditory false recollections to occur across conditions, Misremembered Detail Types were compared within the Multimodal condition in which the number of opportunities for Visual and Auditory false recollections to occur was equal. The Shapiro-Wilk test indicated that the assumption of normality was violated, W = 0.94, *p* = 0.04, and therefore the Wilcoxon rank test was performed. Results showed a significant difference between Auditory and Visual Misremembered Detail Types for the Multimodal narrative condition, Wilcoxon W = 55, *p* < 0.001, rank biserial correlation = -0.95. As shown in Fig 5, the proportion of Visual Misremembered Details was significantly higher than the proportion of Auditory Misremembered Details.

## Discussion

Study 1 showed a memory advantage for complex narratives containing primarily visual details compared to those containing primarily auditory details or both visual and auditory details. Participants showed more accurate gist memory for narratives in the visual condition. Participants also showed more accurate verbatim memory for narratives in the visual condition. Additionally, when broken down by the types of errors made in verbatim memory, proportion of false visual recollections increased with number of auditory details present in the narrative. In contrast, proportion of false auditory recollections did not increase with number of visual details present in the narrative, further suggesting stronger engagement of verbatim memory for visual over auditory information. These findings are consistent with previous research suggesting a bias toward visual information, showing more accurate memory for previously studied pictures of items compared with item labels [17, 18, 26]. We extend this work beyond pictures and labels to show a visual bias in memory for naturalistic, complex events, which may also suggest a bias toward engaging visual imagery when encoding those events.

These findings build upon previous work suggesting that individuals rely heavily on visual information when constructing mental representations of events [27]. Individuals may rely on visual information because of the tight link between visual imagery and episodic memory [16]. There is evidence to suggest that visual imagery supports the recollective experiencing that is characteristic of episodic memory [28], which may facilitate event recall by acting as a structure to guide remembering [14]. It has been suggested that visual images are configural, allowing for easy association between bits of information to effectively retrieve episodic memories [29, 30]. Thus, visual information may be a crucial mechanism by which people piece together episodic details into a coherent narrative. In Study 2, we aimed to further examine this bias toward visual information and conceptually replicate Study 1. We presented participants with auditory only versions of the narratives from Study 1 to more strongly engage imagery processes that may be supporting gist and verbatim memory. Because participants were only presented with the audio unlike in Study 1 in which they received audiovisual cues from the actress, we further chaptered the narratives to reduce demands on long-term memory.

## Study 2

### Methods

#### Participants

Forty-one participants were recruited, however 3 were excluded from data analysis due to having learned English after the age of 5, and 5 were excluded due to technical issues. Thus, the analyzed sample included 33 English speakers (mean age = 21 years old, SD = 1.72 years; 23 female) recruited through McGill University’s participant pool and the Montreal community to participate in this study. All participants reported normal hearing. None of the participants recruited for Study 2 had participated in Study 1. Participants recruited through the Montreal community were given a small compensation for their participation, and those recruited through the participant pool were given course credit.

#### Stimuli and experiment materials

The audio from each video in Study 1 was segmented into 5 chapters per story, with each chapter assigned a title spoken by the same actress who recorded the videos. Participants were presented with these audio-only narratives with chapters separated by chapter titles during the study phase. As in Study 1, none of the participants in Study 2 reported knowing the actress.

Audio-only narratives were presented to participants individually over headphones via Experiment Builder on an ASUS computer running Windows 7. Participants’ free recall of the narratives was recorded using Olympus WS-852 and Sony ICD-PX333 voice recorders. Participants’ subjective ratings of the narratives and responses to questionnaires were collected using Experiment Builder.

#### Design

Like Study 1, Study 2 used a within-subjects design with the independent variables of Narrative Condition (Visual, Auditory, and Multimodal) and Detail Type (Visual, Auditory, and Neutral). The dependent variables were again gist memory, verbatim memory, and false recollections. The Narrative Condition assigned to each narrative and the narrative presentation order was randomized for each participant. Chapter headings for each narrative were consistent across all Narrative conditions.

#### Procedure

All procedures were approved by the McGill University Research Ethics Board and written informed consent was obtained from all participants upon arrival to the lab. Participants were instructed that they would be listening to someone describing several past personal events, and that they would be asked to recall as much as possible from these events. As in Study 1, participants were first presented with a practice phase of the study to become familiar with this task. Participants were presented with the practice narrative (i.e., “Barb works at a store”) via headphones. At the end of the narrative, participants were presented with each chapter title, as heard during the narrative presentations, as a recall cue. Participants then recalled as much as possible from each corresponding chapter while the experimenter recorded their recall using an Olympus WS-852 or Sony ICD-PX333 voice recorder. Following the recall phase, the same subjective ratings from Study 1 were collected for each narrative. Participants repeated this procedure for each experimental narrative.

#### Data analysis

Participants’ recall descriptions were transcribed, scored, and analyzed as in Study 1. Participants’ free recalls in this study were scored by two raters. To establish inter-rater reliability, the two raters first independently scored one participant. Inter-rater reliability was then measured with the intraclass correlation coefficient, giving the correlation between the raters of this participant with values close to one indicating good inter-rater reliability, which was high (ICC = 0.99). After establishing inter-rater reliability, each participant was then scored by one of the two raters for efficiency.

### Results

#### Ratings

One-way repeated measures ANOVAs on each subjective rating showed no significant main effect of Narrative Condition for any of the subjective ratings (emotional valence: *F*(2, 64) = 0.18, *p* = 0.83; emotional intensity: *F*(2, 64) = 0.37, *p* = 0.69; vividness: *F*(2, 64) = 0.17, *p* = 0.84; familiarity: *F*(2, 64) = 0.14, *p* = 0.87). This confirmed that observed effects were not due to differences in participants’ subjective ratings across conditions.

#### Gist memory

**Fig 6:**
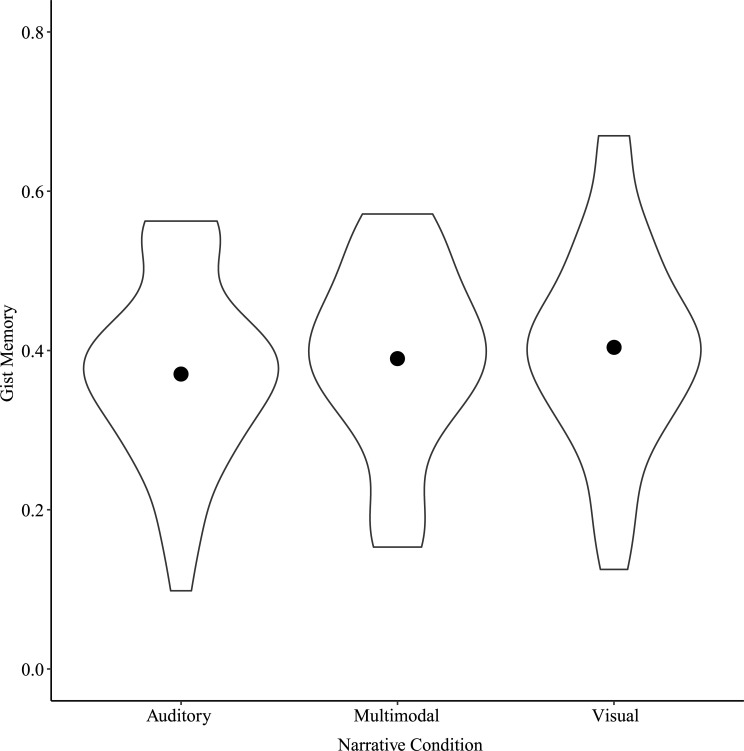
Gist memory by Narrative Condition for Study 2. Points show means for each Narrative Condition.

A one-way repeated measures ANOVA on gist memory scores with Narrative Condition as factor showed a significant main effect of Narrative Condition, *F*(2, 64) = 3.19, *p* < 0.05, η^2^_p_ = 0.09. Post-hoc tests showed a significant difference in gist memory between Auditory and Visual narratives, *t*(64) = -2.52, *p* = 0.04. Gist memory was greater for Visual than for Auditory narratives (Fig 6).

#### Verbatim memory

**Fig 7:**
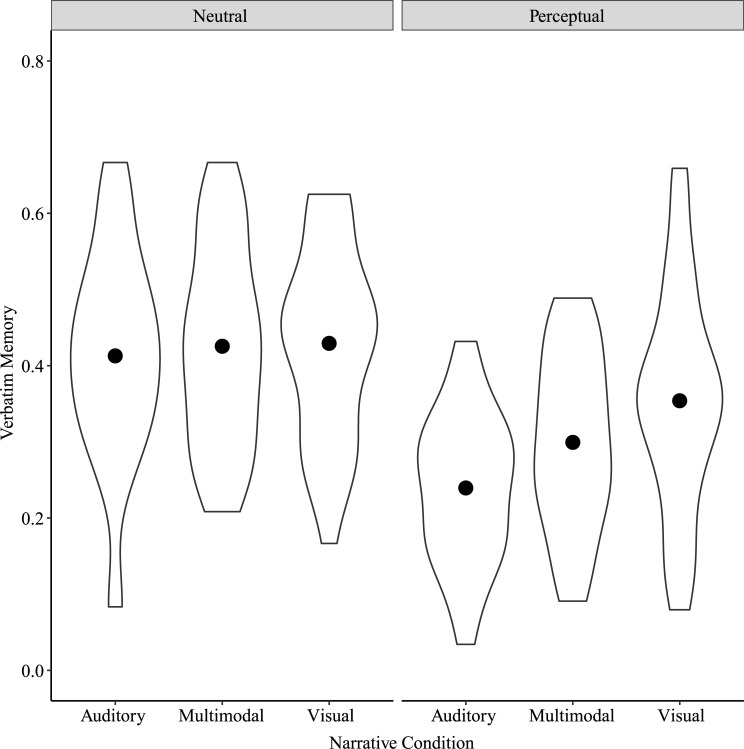
Verbatim memory by Narrative Condition and Detail Type for Study 2. Points show means for each Narrative Condition by Detail Type.

A two-way repeated measures ANOVA on verbatim memory scores with Narrative Condition and Detail Type as factors showed significant main effects of Narrative Condition, *F*(2, 64) = 11.06, *p* < 0.001, η^2^_p_ = 0.26, and Detail Type, *F*(1, 32) = 76.75, *p* < 0.001, η^2^_p_ = 0.71, and a significant interaction between Narrative Condition and Detail Type, *F*(2, 64) = 6.66, *p* = 0.002, η^2^_p_ = 0.17 (Fig 7). Post-hoc tests showed significant differences in verbatim memory for Perceptual details between Auditory and Multimodal narratives, *t*(127.8) = -3.09, *p* = 0.02, Auditory and Visual narratives, *t*(127.8) = -5.91, *p* < 0.001, and Multimodal and Visual narratives, *t*(127.8) = -2.82, *p* = 0.03. Verbatim memory for Perceptual details was greater for Visual narratives than for Multimodal and Auditory narratives, and for Multimodal narratives than for Auditory narratives. There were no differences in verbatim memory for Neutral details by Narrative Condition.

#### False recollections

**Fig 8:**
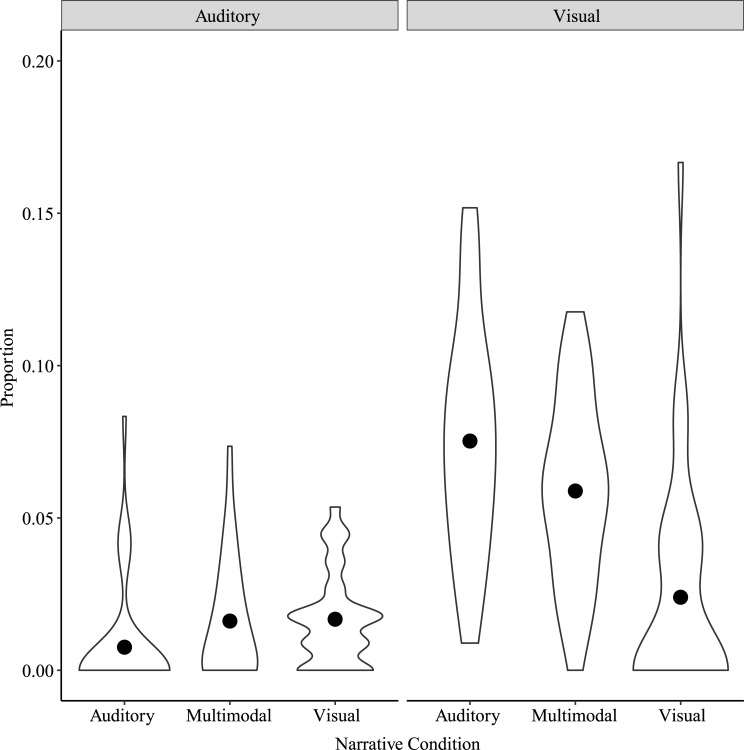
False recollections by Narrative Condition and Misremembered Detail Type for Study 2. Points show means for each Narrative Condition by Misremembered Detail Type. Note the y-axis scale, indicating very few false recollections.

We again focused on Visual and Auditory (i.e., Perceptual) false recollections as in Study 1. A two-way repeated measures ANOVA on false recollections with Narrative Condition and Misremembered Detail Type as factors was conducted. Mauchly’s Test of Sphericity indicated that the assumption of sphericity was violated for the interaction between Narrative Condition and Misremembered Detail Type, Mauchly’s W = 0.73, *p* = 0.008; a Greenhouse-Geisser correction was applied for the interaction. Results showed a significant main effect of Narrative Condition, *F*(2, 64) = 9.67, *p* < 0.001, η^2^_p_ = 0.23, a significant main effect of Misremembered Detail Type, *F*(1, 32) = 126.37, *p* < 0.001, η^2^_p_ = 0.80, and a significant interaction between Narrative Condition and Misremembered Detail Type, *F*(1.58, 50.47) = 19.66, *p* < 0.001, η^2^_p_ = 0.38 (Fig 8). Post-hoc tests showed significant differences for Visual Misremembered Detail Type between Auditory and Visual narratives, *t*(127.7) = 7.30, *p* < 0.001, and Multimodal and Visual narratives, *t*(127.7) = 4.96, *p* < 0.001. Visual Misremembered Details were greatest for Auditory and Multimodal narratives, and smallest for Visual narratives. There were no differences for Auditory Misremembered Detail Type by Narrative Condition.

**Fig 9:**
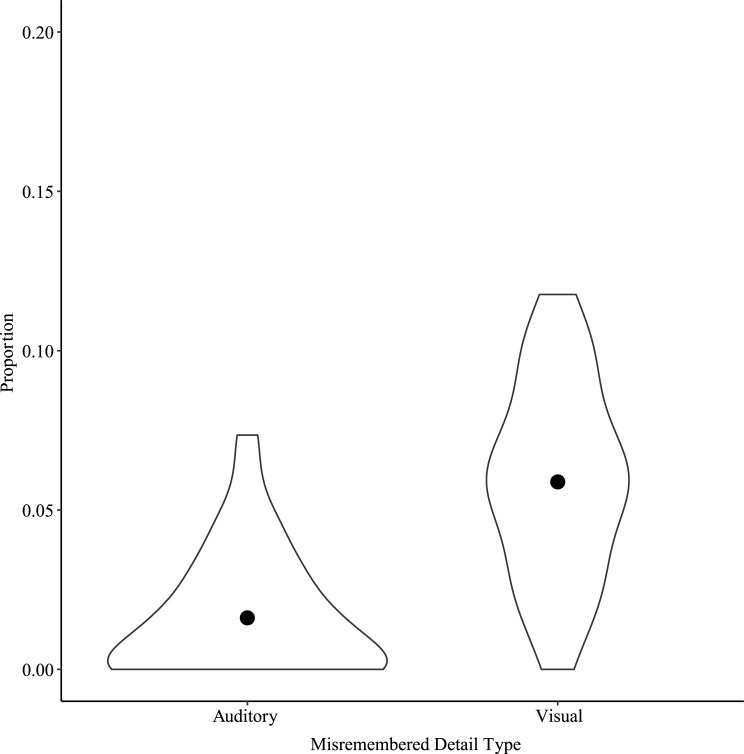
False recollections by Misremembered Detail Type for the Multimodal Narrative Condition in Study 2. Points show means for each Misremembered Detail Type in the Multimodal Narrative Condition. Note the y-axis scale, indicating very few false recollections.

As in Study 1, Visual and Auditory Misremembered Detail Types were compared in the Multimodal Narrative Condition. A paired samples t-test showed a significant difference between Visual and Auditory Misremembered Detail Types in the Multimodal Narrative Condition, *t*(32) = -8.58, *p* < 0.001, Cohen’s d = -1.49. As shown in Fig 9, the proportion of Visual Misremembered Details was significantly higher than the proportion of Auditory Misremembered Details.

## Discussion

Study 2 further investigated the role of visual information in constructing mental representations of complex events at encoding. This study conceptually replicated Study 1 by showing a persistent bias in gist and verbatim memory for events described with primarily visual information. Presenting narratives only in the auditory domain was intended to further engage imagery processes that might be expected to influence how specific details are encoded. However, it is possible that visual imagery processes were able to engage more strongly than auditory imagery processes. Presenting narratives in the auditory domain may have interfered with auditory imagery. Interference has been shown for visual information when presenting dynamic visual noise following a visual stimulus to be kept in memory [31]. Importantly, it has also been shown for auditory information when presenting irrelevant auditory information following an auditory stimulus to be kept in memory [32]. Further research could examine the role of interference during encoding of complex events.

Chaptering the narratives was intended to reduce demands on long-term memory incurred from presenting them only in the auditory domain. It is interesting to note the change in scale between Figs 2 and 6 showing gist memory for Studies 1 and 2, respectively. Participants’ levels of recall in Study 2 appears to have increased compared to Study 1, which could reflect that chaptering the narratives did indeed successfully reduce demands. Interestingly, even while reducing demands, verbatim memory and false recollections still showed the same bias as in Study 1 for events described with primarily visual information.

## General discussion

We conducted two studies to compare how visual, auditory, and non-perceptual information contained within an episodic memory are retrieved, testing the specific hypothesis that visual perceptual details are preferentially recalled from learned episodes when all other factors are controlled. To this end, we constructed naturalistic complex narratives in which the non-perceptual content was controlled while the specific visual and auditory perceptual details were manipulated. In Study 1, we found evidence for a bias toward visual information such that participants had more accurate gist and verbatim memory for events described with more visual than auditory details. We aimed to conceptually replicate and extend these results in Study 2 in which we presented auditory only, chaptered narratives to further engage imagery processes. This was done based on the hypothesized role of imagery processes in helping to encode details of complex events. We replicated Study 1, showing that accuracy of both gist and verbatim memory was greatest when events were described visually.

Overall, our findings provide evidence for a bias toward visual information in encoding of complex narratives even when controlling for other factors (i.e., non-perceptual content). That verbatim memory was more accurate for narratives described with visual information and that false recollections reflected primarily details misremembered with visual information suggests that verbatim memory processes are preferentially engaged by visual information. Interestingly, even gist memory, which does not rely on the precise encoding of specific episodic details, showed this bias. This may reflect the tendency of individuals to activate visual imagery when processing visual information, and the use of more abstract representations when processing auditory information. Neuroimaging work has shown greater activation of medial temporal lobe regions important in memory formation during visual compared with auditory imagery [22]. Research has also indicated that visual imagery is a beneficial method for both encoding and retrieval of information [33, 34]. Our findings fit within this framework suggesting that activating visual imagery processes benefits memory at both general and more fine-grained levels of remembering [for a review, see 35].

A noteworthy methodological choice in our study is that participants heard events that were described to them instead of having firsthand experiences of those events; secondhand descriptions were required to precisely control the event content. Thus, we relied on descriptions to evoke different sensory modalities equally well. It is possible that some details evoked certain sensory modalities more successfully than others. Furthermore, it is possible that additional sensory modalities were evoked beyond those intended, such as sensorimotor when details contained actions. These additional modalities could have been evoked to a greater extent for visual than for auditory details, allowing participants to form more complete mental images and associations between visual episodic details. It is also possible that, although non-perceptual descriptions were constructed so as not to explicitly evoke imaginations of perceptual details (e.g., vision, hearing), participants might have implicitly added these details. This could occur, for example, in the case that the narrator was describing a particular emotion she was experiencing, in which participants might imagine the facial expression corresponding to the emotion described (e.g., visual detail). Future work could examine the extent to which visual or auditory information, along with other perceptual information not considered here, may be implicitly activated when encoding or recalling non-perceptual information. From our study, we would speculate that individuals are biased to activate visual details with non-perceptual information, as suggested by our analysis revealing a tendency to falsely recall details as containing visual information. However, this bias likely varies with individuals, thus future research could also address how individuals vary in the bias to add in perceptual, and more specifically visual, details to non-perceptual content.

Our finding of a bias for visual details within these complex narratives fits with prior reports that indicate that auditory and other nonvisual stimuli are recalled less accurately than visual stimuli [36]. These findings have been interpreted within a source monitoring framework, stating that individuals are better able to discriminate the source of a visual stimulus as being either internally generated or externally presented than an auditory stimulus. Using this framework to interpret our results, it could be that forms of perceptions are recalled at different rates and in different ways (e.g., gist or verbatim memory) as a function of the ability to accurately recollect the source of the percept. In our narratives, perhaps participants were more likely to accurately recall a visual detail than an auditory detail because they could more easily recall the source of the visual detail. Another possibility is that non-visual details require more specificity and thus higher retrieval efforts to recall them from complex events, and thus are not recalled as accurately. This idea follows some work showing that perceptual details, particularly non-visual details, from simulated everyday events are recalled less accurately by older adults who have episodic memory deficits [37]. However, even when retrieval efforts were reduced by “chaptering” the narratives in Study 2, our effect remained.

We did not investigate other factors beyond multimodal interactions that could also shift this bias. Individual differences, including visual and auditory imagery ability or even skill expertise, may shape the way people attend to, perceive, and encode episodic details. Musical expertise has been linked to greater selective auditory attention [38] as well as to structural changes in the hippocampus [39]. Expertise in the visual arts, on the other hand, has been shown to modulate visual attention and has been linked to greater memory for details of studied pictures [40]. Given these findings, it is reasonable to suggest that previous experience and expertise may critically shape the ways in which individuals encode episodic details. Future work could examine this possibility. Additionally, it might have been noted from Table 1 that narratives in the auditory condition sometimes had higher total word counts than in the visual condition. This might suggest that auditory details require more explanation to convey than visual details, a possible confound for the observed findings. However, further analyses with word count as an additional factor did not change the results in either study, suggesting that narrative length cannot account for the visual bias observed here.

In summary, these findings suggest that visual information may play a preferential role in memory for complex, multi-sensory episodic narratives. These findings provide important new directions for future research and real-world applications. Further research is needed to better understand the neural mechanisms underlying preferential treatment of visual information in episodic memory processes, and to understand how other kinds of sensory information can be better utilized by memory systems. With this understanding, new applications and strategies could be developed to aid episodic memory when rich perceptual information is limited or in the case of damage or disease.
